# The Ophthalmic Problem for Hospitals and Doctors

**Published:** 1915-01-23

**Authors:** 


### HOSPITAL WORK IN EGYPT.

### The Ophthalmic Problem for Hospitals and Doctors.

British work and influence in Egypt have just now a special interest, and though it is the political aspect of these which is for the moment particularly prominent, there are other considerations hardly less important. A ruler may be changed, and the political status of the country may be modified, but in the long run the justification of the Government will have to be found in the welfare of the governed. From this point of view, as well as from its own intrinsic interest, the recently issued Report on the Ophthalmic Section of the Department of Public Health, published by the Government Press at Cairo, makes excellent reading. It has been prepared by Dr. A. F. MacCallan, the Director of Ophthalmic Hospitals, who is to be congratulated on an excellent piece of work. The Report illustrates, amongst other conclusions, the benefits which are being conferred on the Egyptian people under the protecting influence of the *Pax Britannica*.

### Egyptian Ophthalmic Hospitals.

The number of ophthalmic hospitals at work in Egypt in 1913 was twelve, and four of these were new hospitals opened in the course of the year. In seven of the principal towns permanent hospitals have been established, and it is hoped shortly to add to this number. Other parts of the country have to be served by travelling hospitals, which go on tour among the smaller towns and are able in this fashion to cover a wide area of service. The scheme of work includes not merely the treatment of patients, but also inspection of school children, lectures on ophthalmic hygiene, and the spread of knowledge in reference to the dangers of infection and the need for cleanliness by pamphlets and by "talks in simple language to collections of women." Such undertakings, of course, mean expense, and for want of money the whole of these proposals are very far from being in operation in all parts of the country. The Report expresses a hope that the next financial year will be more propitious, but since its issue the war has broken out, with, it is to be feared, the necessary postponement, in Egypt as elsewhere, of many beneficial schemes. It is an elementary proposition that the extravagances of war mean the starvation of social reforms.

The surgical staff of the hospitals consists of twenty-one Egyptians, who have obtained their medical diplomas at the Government College in Cairo and have spent not less than two years in the post-graduate study of ophthalmology at the ophthalmic hospitals. Each year a six weeks' course of lectures and practical pathology is given by the Director and the inspecting surgeons. Of the latter, three are English and one an Egyptian. The number of new cases treated was 40,670, the total attendances being 544,267, that is an average of 13.3 visits per patient. Nearly one-third of all the patients treated were under the age of one year.

As is well known, eye diseases are very common in Egypt, and the people are intensely anxious to utilise the ophthalmic relief which is at present available. According to this official Report there is in every small town sufficient clinical material to occupy the whole time of an ophthalmic surgeon. Obviously there are therefore large numbers of patients either wholly untreated or treated by charlatans who "annually spoil thousands of eyes." The Government organisation endeavours to spread a knowledge of ophthalmological work among practitioners by post-graduation courses at the hospitals, and there is also an Ophthalmological Society of Egypt in the annual meeting of which much interest is shown.

### Inspection of Schools.

At the Government Primary School at Tanta, a system of inspection and treatment has been conducted during the last six years. Some 96 per cent. of the children are the victims of trachoma, and the percentage has not substantially varied throughout the period of observation. Infection, it is pointed out, usually occurs during the suckling period, and therefore school treatment can hardly alter the incidence of the disease. It can, however, do much to relieve and improve the individual, and the proportion of contagious cases has been steadily reduced. Subnormal vision is common from opacity of the cornea due in some proportion to trachoma, but more generally the consequence of ulceration associated with acute conjunctivitis. By estimation of the refraction under atropine, and by the prescription of suitable glasses, many children thus affected are given good practical vision, and some 63 per cent. are able to pass the official tests demanded prior to a clerical appointment in the Government service.

### Science and Civilisation.

In the surgical work at the hospitals, 11,700 operations were performed for trichiasis entropion (ingrowing eyelashes), and it is noted that of blind eyes 70 per cent. were due directly or indirectly to various forms of acute conjunctivitis. Much interest attaches to the statement that in primary glaucoma Elliott's operation of trephining has almost entirely superseded the classical iridectomy operation. So much controversy has been conducted on this subject that Dr. MacCallan's experience—a very considerable one—will no doubt be specially noted.

The Report contains many other points of interest and is supported by carefully prepared statistical tables. It is a record of useful and valuable work undertaken in a country for the welfare of which we have a considerable national responsibility. In the discharge of this work Dr. MacCallan is not only contributing to the welfare of his patients, but is, in addition, helping to establish on a firm foundation the civilising mission of British medical science.

